# Simple way of making free-standing cathode electrodes for flexible lithium-ion batteries†

**DOI:** 10.1039/d1ra08993e

**Published:** 2022-03-24

**Authors:** Chih-Hung Chen, Jian-Ming Chiu, Indrajit Shown, Chen-Hao Wang

**Affiliations:** Department of Materials Science and Engineering, National Taiwan University of Science and Technology Taipei 106335 Taiwan chwang@mail.ntust.edu.tw; Department of Chemical Engineering, National Taiwan University of Science and Technology Taipei 106335 Taiwan; Department of Chemistry, Hindustan Institute of Technology and Science Chennai 603103 India; Center of Automation and Control, National Taiwan University of Science and Technology Taipei 106335 Taiwan

## Abstract

The flexible electrodes used in the lithium-ion battery (LIB) offer an excellent opportunity to be bent and folded without deforming their electrochemical characteristics. However, a flexible electrode does not include metal foil as a current collector, limiting the LIB's flexibility and weakening the mechanical strength. This study fabricates flexible LiFePO_4_ (LFP) free-standing electrodes by a scalable and straightforward solution-based etching process. The obtained free-standing electrodes show capacities and bending performances that are similar to the conventional electrodes with aluminum current collectors. This study opens a new avenue for developing a free-standing electrode for low-cost and flexible lithium-ion batteries.

## Abstract 2

The flexible electrodes used in the lithium-ion battery (LIB) offer an excellent opportunity to be bent and folded without deforming their electrochemical characteristics.

## Graphical abstract

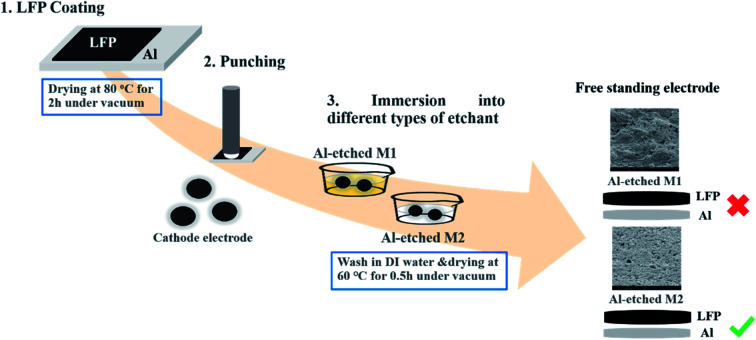

## 1. Introduction

In recent years within a short period, the demand for flexible lithium-ion batteries (LIBs) with high-energy capacity vastly increased due to the rapid development of foldable and wearable devices such as roll-up displays, active radio-frequency identify cation tags, and integrated circuit smart cards.^1–3^ LIBs can be flexible or even foldable *via* the conventional engineering approach by making thinner ones, but LIBs with lighter configurations will significantly reduce the overall capacity. Therefore, different methods need to develop to manufacture flexible and high-capacity LIBs.^4,5^ Conventional LIB metal current collectors such as copper (Cu) and aluminum (Al) are typically used as anode and cathode, respectively. Moreover, these rigid and non-flexible metal current collectors have been considered dead weight in LIBs. Hence they significantly reduce the overall energy density of LIBs and limit their flexibility.^6–8^

In a new approach to fabricate lighter, more secure, and more efficient LIBs, a variety of carbon-based current collectors, including carbon nanotubes,^9–11^ carbon paper,^12–14^ graphene paper,^15–17^ and carbon fiber^18,19^ have been developed to replace the traditional metal foils. For instance, Chen *et al.* developed a highly conductive (∼3000 S cm^−1^) reduced graphene oxide film by the current-induced annealing method and demonstrated its applicability as a lightweight current collector.^20^ Liu *et al.* reported TiO_2_ and activated carbon-fiber composite as light and ultra-long life (2000 cycles at 20C) of LIB performance where the intrinsic open channel structure and large surface area of the ultra-thin TiO_2_ nano-sheets possessed the fast charge and discharge.^21^ However, the higher resistance of the electrodes and lower loading level of the active material of the previous approaches have created a significant obstacle for making flexible LIBs. Moreover, the previously reported methods require a modified fabrication process and an extensive investigation of the reliability before the flexible applications. Practically, suppose we could re-engineer one of the LIBs components (metal foil current collectors) with the free-standing flexible electrodes. In that case, it immediately overcame all the fabrication issues of efficient LIBs. Therefore, we believe further investigation and the feasibility study of utilizing free-standing flexible electrodes for LIBs which are necessary for the configuration of flexible LIBs.

Herein, in this study, we prepare a free-standing and flexible electrode (cathode) by a conventional fabricating process with LiFePO_4_ (LFP) for LIBs to maximize the reliability for flexible device application. Additionally, we introduce two different etchants for the as-prepared flexible electrode compared to the physical and electrochemical properties of the cathode material. This novel lightweight free-standing cathode electrode possesses an extremely high energy density than the conventional LFP/Al electrode for flexible LIB application.

## 2. Experimental section

### 2.1. Materials

Polyvinylidene fluoride (PVDF) and LFP were purchased from Union Chemical Ind. Co., LTD. Super P and *N*-methyl-2-pyrrolidone (NMP) solvents were purchased from Alfa Aesar. Al foil was purchased from Ubiq Tech Co., Ltd. Two etchants, H_3_PO_4_-based etchant (Al Etch-M1) and TMAH-based etchant (Al Etch-M2), were obtained from Hai-Bo Advanced Chem-Materials Co., Ltd.

### 2.2. Preparation of free-standing electrode

**Fig. 1:**
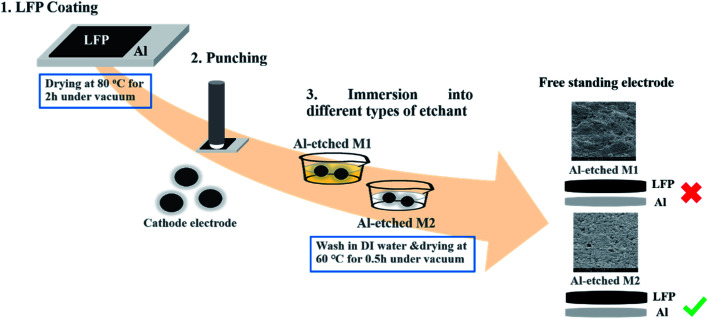
Schematic illustration of free-standing LFP/Al electrode fabrication steps.

The slurry was composed of LFP (80%) with PVDF (10%) and Super P (10%) in NMP, then coated on carbon-coated aluminum foil and dried in a vacuum at 120 °C for 12 h. The thickness and loading levels were 36 μm and 1.65 mg cm^−2^, respectively. To prepare the flexible LFP free-standing film, the prepared LFP/Al film electrode was directly placed in two different undiluted etchants: Al Etch-M1 etchant and Al Etch-M2 etchant to etch the Al foil within 1 minute at 25 °C, respectively. All the chemicals and solvents are used at their original concentrations without further dilution. Finally, the free-standing LFP film was collected from the respective etchants, washed with DI water, and dried at 60 °C for 30 min under vacuum. The complete schematic preparation route for LFP free-standing electrode (LFP-Al) is illustrated in Fig. 1. For comparison study, a conventional cathode with carbon-coated Al-foil current collector was also prepared by using a similar slurry solution composed of LFP (80%) with PVDF (10%) and Super P (10%) in NMP.

### 2.3. Characterizations

A field-emission scanning electron microscope (FE-SEM, JSM-6500) analyzed all samples' surface morphology and cross-section image. All samples' short-range order crystal structure from Raman spectra were obtained using MRS5000 Micro Raman Spectrometer 532 nm with 125 mW DPSS laser (Rayleytek Co., Ltd). Different etchant solutions were determined iron (Fe) using inductively coupled plasma optic emission spectrometry (ICP-OES, Spectro Arcos, SPECTRO model Analytical Devices, PerkinElmer OPTIMA-7300DV). First, the etchant solution was dried on the dish, and then add the 6% HNO_3_ to cover the samples. Finally, the solution was injected into the vial and lid on the ICP-OES for detection. Typically, the samples prepared for the iron dissolution experiments were injected into an air/acetylene flame. After 10 s of stabilization, the measurement was recorded. In ICP-OES, three measures (5 s each) were recorded for iron absorbance at the wavelength of 372 nm. The iron contents of the samples were obtained from the calibration curve of various Fe concentrations prepared by using an iron standard solution (Sigma-Aldrich [Fe] = 1000 ppm).

### 2.4. LIB fabrication

The LFP/Al electrode and LFP free-standing electrode were introduced in the typical CR2032 coin-type cells. In the CR2032 coin-type cells fabrication, lithium metal was used as a counter electrode. As an electrolyte, 1 M LiPF_6_ in a mixture of 1 : 1 (v/v) ethylene carbonate (EC) and diethyl carbonate (DEC) was used as a counter electrode and an electrolyte, respectively. A Celgard 2325 triple layer of PP/PE/PP membrane was used as a separator was soaked in electrolyte for 24 h before cell assembly. The cells were assembled inside an Ar-filled glove box where the H_2_O and O_2_ were lower than 1 ppm. The charge–discharge cycling properties of the as-prepared CR2032 coin-type cells were measured in the voltage region of 2.5–4.2 mV (*vs.* Li/Li^+^) under a constant current using an Ubiq BAT-750B battery test system. After fabricating the LFP/Al and LFP free-standing film battery, all battery coin-cells were stored for 8 h before the charging-discharging process for stabilization. The cyclic voltammogram (CV) was performed at a scan rate of 0.1 mV s^−1^ over a range of 2.5 to 4.2 mV (*vs.* Li/Li^+^) on the Autolab electrochemical station. The electrochemical impedance spectroscopy measurements were also carried out from 0.1 Hz to 100 kHz using a 10 mV AC signal.

## 3. Results and discussion

### 3.1. Characterization

**Fig. 2:**
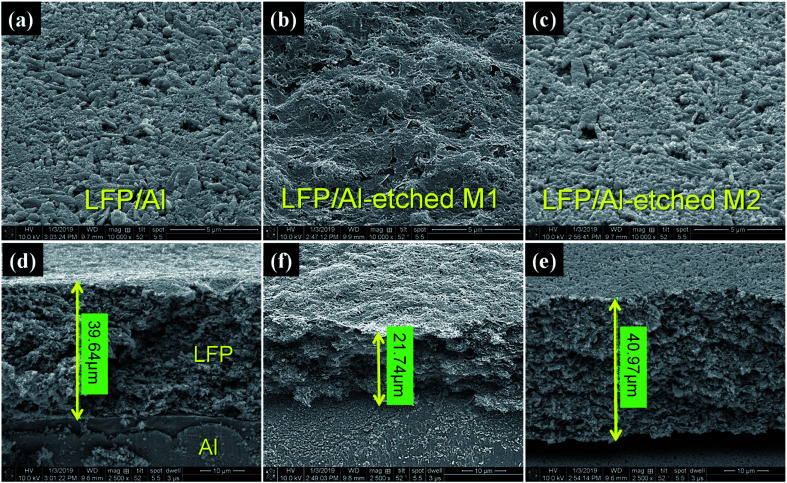
SEM images of the (a–c) surface morphology (top-view) and (d–f) cross-section view of LFP/Al electrode, LFP/Al-etched M1 electrode, and LFP/Al-etched M2 electrode, respectively.

Field-emission scanning electron microscopy (FE-SEM) was used to clarify the microstructure differences on the surface and cross-section of all prepared LFP/Al electrodes before and after etching processes, as shown in Fig. 2. The top-view surface morphology images of Fig. 2a–c show that the LFP particles are well dispersed and bounded with PVDF and SP. The surface morphology of LFP/Al-etched M1 electrode clearly shows the interwoven network and smooth structure. In contrast, LFP/Al electrode and LFP/Al-etched M2 electrode surfaces only have small inter-particle connected void space. The observed pores and voids between LFP particles can provide channels for effective electrolyte diffusion and absorption, which possesses an essential impact on the electrochemical performance for improving the rate performance of the battery. In Fig. 2d and e, the cross-sectional images of LFP/Al electrode and LFP/Al-etched M2 show nearly the same LFP layer thickness of around 39.6 μm and 40.97 μm, respectively. For comparison, the LFP/Al-etched M1 electrode shows 21.74 μm of LFP layer thickness, nearly half of the LFP/Al electrode and the LFP/Al-etched M2 electrode. Therefore, the Al Etch-M1 etchant shows a higher etching rate than the Al Etch-M2 etchant, indicating that the Al Etch-M2 etchant is better at fabricating the flexible free-standing electrode.

**Fig. 3:**
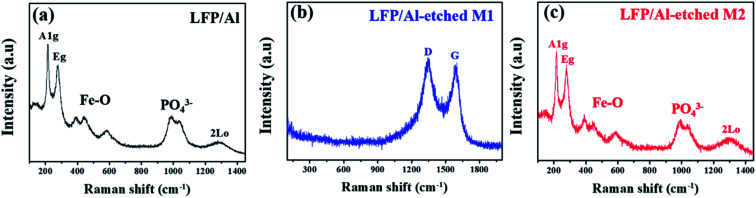
Raman spectra of (a) LFP/Al, (b) LFP/Al-etched M1, and (c) LFP/Al-etched M2.

Fig. 3 shows Raman spectroscopy about the structural transformation of LFP electrodes during the etching process in the presence of different etchants. LFP/Al electrode and LFP/Al-etched M2 electrode show two typical characteristic A1g and Eg band vibrations of –Fe_2_O_3_ at about 217 and 277 cm^−1^, as shown in Fig. 3a and c.^22–24^ Additionally, the other observed bands approximately in the region from 300–1100 cm^−1^ correspond to the intra-molecular stretching modes of the specific –Fe–O (300–600 cm^−1^) and –PO_4_ (900–1100 cm^−1^) of LFP. The observed broad peak of PO_4_ stretching bands is well matched with the other reports where three different regions appear at 950 cm^−1^, 986 cm^−1^, and 1058 cm^−1^, respectively.^25,26^ Furthermore, LFP/Al electrode and LFP/Al-etched M2 electrode show broadband at around 1280–1300 cm^−1^, related to the second-order disorder band of Fe_2_O_3_ (2LO).^27^ LFP/Al-etched M2 electrode shows similar bands as LFA/Al, revealing that the Al-etched M2 etchant retains the LFP layer without any chemical decoration. However, the Raman spectrum of LFP/Al-etched M1 electrode clearly shows only two specific bands around 1350 to 1580 cm^−1^ for D and G band of carbon without any particular peaks of LFP, as shown in Fig. 3b, indicating that the Al Etch-M1 etchant destroys the LFP crystals. Only amorphous carbon slag is left as an underline layer.

To understand the in-depth etchants for the Al etching process, the iron contents of the etched solutions from the Al Etch-M1 etchant and the Al Etch-M2 etchant were monitored using inductively coupled plasma optical emission spectrometry (ICP-OES). The iron contents in the 10 min etched solutions are summarized in Table 1. The etched solutions from the Al Etch-M2 etchant show Fe content around 8 ppm and 24 ppm for 10 min and 40 min etching process, respectively. When the LFP/Al immerse into the Al Etch-M1 etchant, a significant etching reaction occurs; therefore, an increased Fe content value is observed around 25 521 ppb and 26 380 ppb for 10 and 40 min etching process, respectively. Thus, LFP/Al-etched M2 electrode shows excellent selectivity of Al etching under the Al Etch-M2 etchant without affecting the Fe content of the active LFP in the electrode.

**Table 1:** Fe contents of the etched solutions with different etchants

Electrode	Etchant	Fe content (ppb) after 10 min	Fe content (ppb) after 40 min
LFP/Al-etched M1	Al Etch-M1	25 521	26 380
LFP/Al-etched M2	Al Etch-M2	8	24

### 3.2. LIB performance

**Fig. 4:**
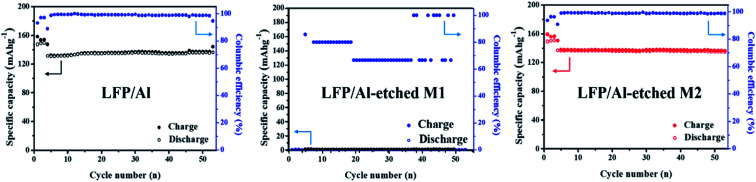
Cycling stabilities of LIBs using LFP/Al electrode, LFP/Al-etched M1 electrode, and LFP/Al-etched M2 electrode.

To understand the various factors contributing to electrode performances, Fig. 4 shows the LIB performances of the cell using LFP/Al electrode, LFP/Al-etched M1 electrode, and LFP/Al-etched M2 electrode. The initial three cycles were carried out at 0.1C and the following 50 cycles at 0.5C. The batteries using LFP/Al electrode and LFP/Al-etched M2 electrode show stable cycling up to 50 cycles, as shown in Fig. 4a and c, respectively. They exhibit excellent coulombic efficiencies over 99% after the second cycle. However, the LIB using LFP/Al-etched M1 electrode demonstrates almost zero capacity. The observed battery cycle performance reveals that the deterioration in the LFP crystal structure during the Al Etch-M1 etching affects the performance of free-standing FEA/Al-etched M1 electrodes.

**Fig. 5:**
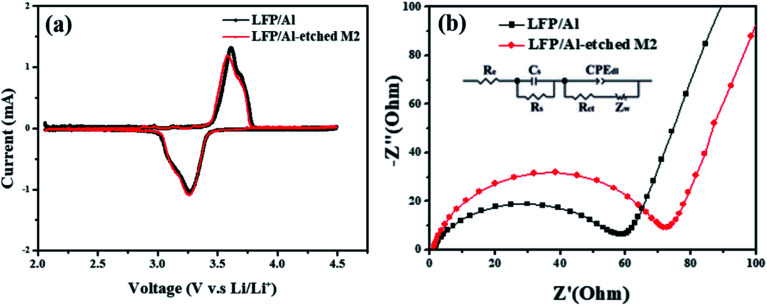
(a) Cyclic voltammograms of LFP/Al electrode and LFP/Al-etched M2 electrode from 2.0 V to 4.5 V at a scan rate of 0.1 mV s^−1^; (b) EIS results for LFP/Al electrode and LFP/Al-etched M2 electrode.

The free-standing cathode's LFP/Al-etched M2 capacity is between 135 and 137 mA h g^−1^. In comparison, the supported electrode capacity drops from 137 mA h g^−1^ of C-LFP to only 56 mA h g^−1^ of the electrode (empty symbols in Fig. S4†). The collector-free demonstrated here leads to an apparent increase of the gravimetric specific capacity by reducing the amount of inert material within the entire electrode. Additionally, to evaluate the electrochemical performance, the cyclic voltammetry (CV) between 2.0–4.5 V (*versus* Li^+^/Li) at a scan rate of 0.1 mV s^−1^ was conducted using 1 M LiPF_6_ in 1 : 1 (v/v) EC/DEC as an electrolyte, as shown in Fig. 5a. The CV curves for LFP/Al-etched M2 electrode (black line) and LFP/Al electrode (red line) indicate that the Al Etch-M2 etchant does not adversely affect the electrochemical properties of the LFP electrode because the polarization (Δ*E*) of the two electrodes are similar. Furthermore, the observed potential gap changed from 0.34 V to 0.31 V for the LFP/Al-etched M2 electrode, smaller than 0.03 V than the LFP/Al electrode, indicating that LFP/Al-etched M2 electrode has a better kinetics process and stability than LFP/Al electrode. The electrochemical impedance study was performed in Fig. 5b. The Nyquist plot shows an intercept on the real axis at high frequencies attributed to the electrolyte resistance. A semicircle was observed in the high-middle frequency region for two electrodes. The diameter of this semicircle on the *Z*_re_ axis is approximately equal to the charge-transfer resistance through the electrode/electrolyte interface. The observed straight line at the lower frequency region reveals the diffusion of lithium ions into the bulk of the electrode. The calculated fitting values of the charge-transfer resistance *R*_ct_ of LFP/Al electrode is 59 Ω, which is higher than that of LFP/Al-etched M2 electrode (*R*_ct_ = 73 Ω). The lower *R*_ct_ value is attributed to the better charge transfer characteristics for LFP/Al-etched M2 electrode than LFP/Al electrode. Notably, the LFP/Al-etched M2 electrode is more flexible and lighter than the LFP/Al electrode. After carefully evaluating all the results, LFP/Al-etched M2 electrode fabricated by a novel method could be an excellent candidate for highly flexible LIBs.

### 3.3. Bending stress test

**Fig. 6:**
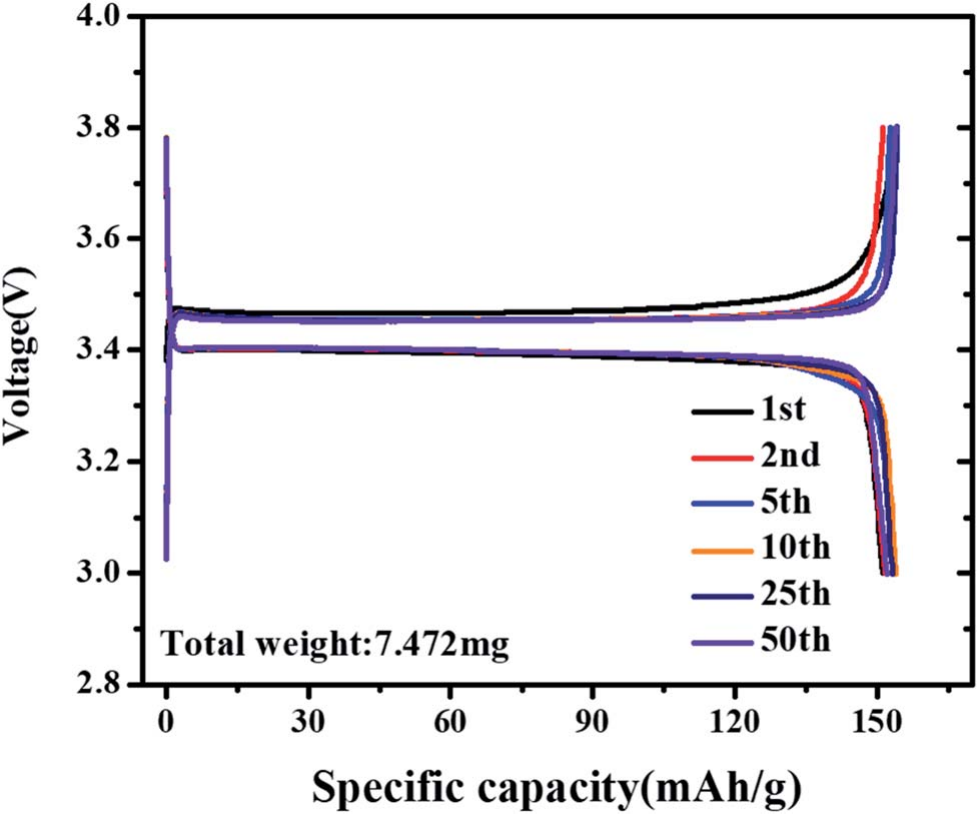
Charge–discharge profiles of after bending stress tests using LFP/Al-etched M2 electrode.

To investigate the cycling stability of the cell under bending stress for LFP/Al-etched M2 electrode, the galvanostatic charging/discharging curves of the LIB are assembled after the electrode bending condition (50 times at bending radius 3 mm), as shown in Fig. 6. The initial discharge capacity of the battery is around 154 mA h g^−1^ with coulombic efficiency of 98% at 0.2C. The detail of capacity changes at different cycles is shown in Table 2. The observed galvanostatic charging/discharging curves under bending stress reveal the superior flexibility and electrochemical performance of LFP/Al-etched M2 electrode.

**Table 2:** The cycle capacity summarized from Fig. 6

Cycles	Cycle capacity (mA h g^−1^)	
	Discharge	Charge
1st	151.07	154.08
2nd	151.53	151.16
5th	153.22	152.78
10th	153.84	154.01
25th	153.17	154.19
50th	152.08	153.80

### 3.4. Mechanism insight of different etchant

The wet chemical aluminum etching process using the Al Etch-M1 etchant a well-established for many electronics industries.^28^ The functions of each component in this specific etchant are different, like the nitric acid used for alumina oxidation reaction; phosphoric acid removes the reaction products away from the surface of the substrate, and acetic acid is used as a diluent to maintain a low etching concentration. The primary mechanism of wet chemical aluminum etching reaction steps are as follows:^29^

Anode:Al → Al^3+^ + 3e^−^

Cathode:O_2_ + 2H_2_O + 4e^−^ → 4OH^−^2H^+^ + 2e^−^ → H_2_

Precipitation reaction of metal hydroxides:Al^3+^ + 3OH^−^ → Al(OH)_3_

Alumina or aluminum hydroxide dissociates as trivalent aluminum ions in acidic solutions with a pH less than 4. The reactions steps are as followsAl(OH)_3_ + 3H_3_O^+^ → Al^3+^ + 6H_2_OAl_2_O_3_ + 6H_3_O^+^ → 2Al^3+^ + 9H_2_O

The as-prepared lightweight LFP/Al-etched M1 electrode was used as the cathode electrode for the LFP battery. However, the fabricated battery based on LFP/Al-etched M1 electrode fails to perform the charging and discharging process during galvanostatic charge–discharge measurement. The Al Etch-M1 etchant contains a certain percentage of HNO_3_; therefore, the highly oxidative HNO_3_ effectively attacks the Fe in the LFP electrode material under an acidic medium followed by oxidation, hydrolysis, and crystallization reaction iron convert into hematite.^29^ The complete reaction steps are as follows.^30^

Oxidation:4Fe + 10HNO_3_ → 4Fe(NO_3_)_2_ + NH_4_NO_3_ + 3H_2_O3Fe^2+^ + 4H^+^ + NO_3_^−^ → 3Fe^3+^ + NO↑ + 2H_2_O

Hydrolysis:Fe^2+^ + H_2_O → [FeOH]^2+^ + 4H^+^[FeOH]^2+^ + H_2_O → [Fe(OH)_2_]^+^ + H^+^2Fe^3+^ + H_2_O → [Fe_2_(OH)_2_]^4+^ + 2H^+^

Crystallization or nucleation:5Fe_2_(OH)_2_^4+^ + 14H_2_O → 5Fe_2_O_3_·9H_2_O + 20H^+^

In acidic solutions, the formation of hematite:5Fe_2_O_3_·9H_2_O + 30H^+^ + 36H_2_O → 10[Fe(H_2_O)_6_]^3+^2Fe(H_2_O)_6_^3+^ → Fe_2_O_3_ + 9H_2_O + 6H^+^

On the other hand, the Al Etch-M2 etchant shows high selectivity without affecting the iron content of the LFP cathode material. The etching mechanism of the Al Etch-M2 etchant is as follows.^30^

Anode:Al → Al^3+^ + 3e^−^

Cathode:O_2_ + 2H_2_O + 4e^−^ → 4OH^−^2H^+^ + 2e^−^ → H_2_

Precipitation reaction of metal hydroxides:Al^3+^ + 3OH^−^ → Al(OH)_3_

Since the Al Etch-M2 etchant is an alkaline solution, alumina or aluminum hydroxide dissolves and form the aluminum complexion, as follows:Al(OH)_3_ + OH^−^ → Al(OH)_4_^−^Al_2_O_3_ + 3H_2_O + 2OH^−^ → 2Al(OH)_4_^−^

Abderrahim *et al.* demonstrated that 3 wt% TMAH is a corrosion inhibitor for stainless steel (iron-rich).^31^ Likewise, Kuhn *et al.* reported that the TMAH solution was highly selective for iron corrosion when used as a desalting agent for archaeological iron artifacts.^32^ After the Al Etch-M2 etchant etches the electrode, the LIB using LFP/Al-etched M2 electrode has no degradation after 50 charge–discharge cycles, indicating that the Al Etch-M2 etchant only etches aluminum.

## 4. Conclusion

This study demonstrates the successful fabrication of a high-energy-density battery based on a free-standing and flexible electrode by using a simple etching process to remove aluminum foil. This free-standing LFP/Al-etched M2 electrode has good flexibility, high capacity, and stable cyclic performance even under the bending stress tests. Moreover, this simple etching technique is versatile for fabricating a broad class of flexible free-standing anode and cathode materials for LIBs, which is applied in portable or wearable electronic devices.

## Conflicts of interest

There are no conflicts to declare.

## Supplementary Material
